# Hyperphosphatemia and its relationship with blood pressure, vasoconstriction, and endothelial cell dysfunction in hypertensive hemodialysis patients

**DOI:** 10.1186/s12882-022-02918-0

**Published:** 2022-08-23

**Authors:** Jinwoo Jung, Haekyung Jeon-Slaughter, Hang Nguyen, Jiten Patel, Kamalanathan K. Sambandam, Shani Shastri, Peter Noel Van Buren

**Affiliations:** 1 Department of Internal Medicine, Division of Nephrology, University of Texas Southwestern Medical Center, 5939 Harry Hines Boulevard, Dallas, TX USA; 2 Medical Service, Dallas Veterans Affairs Medical Center, Dallas, TX USA; 3 Southern Methodist University, Dallas, TX USA

**Keywords:** Phosphate, Hemodialysis, Endothelial cell dysfunction, Mineral bone disease, Vasoconstriction

## Abstract

**Background:**

Hyperphosphatemia occurs frequently in end-stage renal disease patients on hemodialysis and is associated with increased mortality. Hyperphosphatemia contributes to vascular calcification in these patients, but there is emerging evidence that it is also associated with endothelial cell dysfunction.

**Methods:**

We conducted a cross-sectional study in hypertensive hemodialysis patients. We obtained pre-hemodialysis measurements of total peripheral resistance index (TPRI, non-invasive cardiac output monitor) and plasma levels of endothelin-1 (ET-1) and asymmetric dimethylarginine (ADMA). We ascertained the routine peridialytic blood pressure (BP) measurements from that treatment and the most recent pre-hemodialysis serum phosphate levels. We used generalized linear regression analyses to determine independent associations between serum phosphate with BP, TPRI, ET-1, and ADMA while controlling for demographic variables, parathyroid hormone (PTH), and interdialytic weight gain.

**Results:**

There were 54 patients analyzed. Mean pre-HD supine and seated systolic and diastolic BP were 164 (27), 158 (21), 91.5 (17), and 86.1 (16) mmHg. Mean serum phosphate was 5.89 (1.8) mg/dL. There were significant correlations between phosphate with all pre-hemodialysis BP measurements (*r* = 0.3, *p* = .04; *r* = 0.4, *p* = .002; *r* = 0.5, *p* < .0001; and *r* = 0.5, *p* = .0003.) The correlations with phosphate and TPRI, ET-1, and ADMA were 0.3 (*p* = .01), 0.4 (*p* = .007), and 0.3 (*p* = .04). In our final linear regression analyses controlling for baseline characteristics, PTH, and interdialytic weight gain, independent associations between phosphate with pre-hemodialysis diastolic BP, TPRI, and ET-1 were retained (β = 4.33, *p* = .0002; log transformed β = 0.05, *p* = .005; reciprocal transformed β = -0.03, *p* = .047).

**Conclusions:**

Serum phosphate concentration is independently associated with higher pre-HD BP, vasoconstriction, and markers of endothelial cell dysfunction. These findings demonstrate an additional negative impact of hyperphosphatemia on cardiovascular health beyond vascular calcification.

**Trial registration:**

The study was part of a registered clinical trial, NCT01862497 (May 24, 2013).

## Introduction

Mineral bone disease (MBD) is a non-traditional cardiovascular disease risk factor that contributes to the high mortality rate among end stage renal disease (ESRD) patients on hemodialysis (HD). Hyperphosphatemia and the related increases in parathyroid hormone (PTH) are each independently associated with mortality in this population [1]. Chronically elevated serum phosphate contributes to structural changes in blood vessels including vascular calcification, but it is also likely to be associated with other *functional* abnormalities of the blood vessels that may be more easily reversible.

Recent experimental evidence shows that acute increases in phosphate both in vitro and in healthy individuals can induce acute endothelial cell dysfunction (ECD) [2]. The most common clinical scenario where hyperphosphatemia is observed is in advanced chronic kidney disease, especially ESRD. Compared to healthy individuals, ESRD patients also have a much higher risk of hypertension and more severe ECD [3, 4] with ECD markers such as asymmetric dimethylarginine (ADMA) being predictive of increased mortality [5]. Despite this, there is little data on the relationship between phosphate and adverse cardiovascular consequences beyond vascular calcification. A better understanding of these relationships is needed to provide a more comprehensive approach to addressing mortality risk reduction in ESRD patients.

We hypothesized that elevated serum phosphate would be independently associated with blood pressure (BP), as well as measurements of vasoconstriction and markers of ECD in hypertensive hemodialysis patients. We analyzed these associations within a cohort of patients receiving maintenance HD.

## Materials and methods

### Study participants

We used baseline data from a previously conducted prospective cohort of hypertensive ESRD patients on maintenance HD patients which included a predefined subset with recurrent intradialytic hypertension [6]. Study inclusion criteria were 1) age > 18 years, 2) HD vintage > 1 month, and 3) peri-dialytic hypertension defined as pre-HD systolic BP > 140 mmHg or post-HD systolic BP > 130 mmHg [7]. Exclusion criteria (largely due to the use of bioimpedance spectroscopy from the prior study) were defined by the presence of a cardiac defibrillator or pacemaker, arm or leg amputation, coronary artery stent, implanted metallic prosthesis, pregnancy, or the inability to achieve estimated dry weight defined by the nephrologist providing the clinical care. The University of Texas Southwestern Medical Center Institutional Review Board approved the protocol, and all procedures were in accordance with the Declaration of Helsinki. The study was part of a registered clinical trial, NCT01862497 [8] registered May 24, 2013.

### Study procedures

Before and after a mid-week HD treatment we obtained TPRI, BP, ET-1 and ADMA measurements detailed below:

#### Impedance cardiography

We obtained measurements 20 min prior to and 20 min following a mid-week hemodialysis treatment. Using impedance cardiography (Non-Invasive Cardiac Output Monitor [NICOM], Cheetah Medical Inc, Newton Center MA) we placed electrodes on the anterior and posterior trunk bilaterally. With the participant in the supine position, we simultaneously measured brachial artery systolic and diastolic BP and cardiac output. Three measurements were obtained at one-minute intervals, and the mean value was calculated. The total peripheral resistance (TPR) was automatically calculated by the device using the formula: mean arterial pressure (MAP) = TPR x cardiac output (CO) and expressed as dynes*seconds/cm^5^. The TPRI was automatically calculated per body surface area using the height and weight obtained in the dialysis unit.

#### Peridialytic blood pressure measurements

Immediately prior to and after the same mid-week hemodialysis treatment, dialysis unit staff measured seated systolic and diastolic BP according to dialysis unit standard procedures using sphygmomanometers attached to the hemodialysis machine. These measurements were automatically recorded in the electronic medical record.

#### Ambulatory blood pressure

Immediately following the mid-week treatment, we began ambulatory BP measurements with a Spacelabs 90207 machine. The first reading was obtained in the hemodialysis unit, and subsequent measurements were obtained every 30 min during the daytime and every hour from 10 pm to 6 am for 44 h until the next dialysis treatment.

#### Laboratory data

We obtained the most recent standard HD laboratory measurements from the patient’s electronic medical record that include pre-HD measurements of hemoglobin, serum phosphate, serum creatinine, serum albumin, serum PTH, serum calcium, blood urea nitrogen, and protein catabolic rate (PCR). Immediately prior to the mid-week treatment in which BP and TPRI were measured, we obtained additional plasma for measurements of endothelin-1 (ET-1) and ADMA. These samples were stored in a -80 degree C freezer and were analyzed in batch using quantitative sandwich enzyme immunoassay technique with Human Endothelin-1 Immunoassay (Quantiglo) for ET-1 and competitive enzyme linked immune-sorbent assay (Biovendor) with a microtiter plate format for ADMA.

#### Statistical analysis

We reported descriptive statistics as mean and standard deviations for continuous variables and frequencies and percentages for categorical variables. Data determined to be a significant outlier (n = 1) was not included in final analysis.

We used Pearson and Spearman correlations to test whether serum phosphate are related to study outcome BP variables, other continuous variables related to BP, and laboratory measurements. When relations are significant, we then used serum phosphate as the primary predictor variable in generalized linear regression models (GLM) with the following outcome variables described in detail below (systolic and diastolic BP, pre-HD TPRI, pre-HD ET-1, pre-HD ADMA) controlling for confounders.

Because the peridialytic BP measurements were related to each other for each participant, our models using BP as the outcome variable were repeated-measures linear regression analysis where the initial supine BP measurement (20 min pre-hemodialysis) was the primary outcome variable. We conducted separate analyses for systolic and diastolic BP. In Model 1, we controlled for age, sex, race/ethnicity, diabetes, and the remaining peri-dialytic BP measurements. We combined race and ethnicity into a binary variable: non-Hispanic White and non-white. This coarsening of race and ethnicity data into one single variable is accepted because 1) all participants were either non-Hispanic white, Hispanic white, or non-Hispanic Black and 2) both Black race and Hispanic ethnicity have been associated with higher pre-hemodialysis systolic BP [9]. In Model 2, we also included PTH and interdialytic weight gain (expressed as a percentage of weight) as independent variables.

For the remaining outcome variables (pre-HD TPRI, ET-1 and ADMA), we used separate GLM with serum phosphate as the primary predictor variable. To normalize the distributions, appropriate data transformations were completed (logarithmic transformation for TPRI and ADMA; reciprocal transformation for ET-1). We controlled for age, sex, race/ethnicity, and diabetes. We used Loglikelihood Ratio test and Akaike Information Criteria (AIC) for goodness of fit tests and statistical significance to determine inclusion and exclusion of covariates in the final model. Our final model (model 2) also controlled for PTH and interdialytic weight gain because this had the lowest AIC, but we also explored other models that included serum calcium, albumin, and PCR. A p-value < 0.05 as set as a criterion for statistical significance. All statistical analyses were conducted using SAS 9.4 version (SAS Institute, Cary, NC).

## Results

### Participant characteristics

From a cohort of 75 patients, there were 54 with complete data available for serum phosphate, the outcomes of pre-HD BP, TPRI, serum ET-1 and ADMA, and other analyzed covariates. Thus, a final study sample size is 54. The characteristics of these 54 patients are depicted in Table 1. Sixty-one percent of the participants were male, and 63% had diabetes. Almost all participants were receiving at least one oral phosphate binder (56% on calcium-containing binders, 41% on non-calcium-containing binders). There were 80% receiving some form of active vitamin D, and 33% receiving cinacalcet. The phosphate levels were collected from a range of 0–32 days from the time the BP, TPRI, ET-1, and ADMA measurements were obtained (median 10 [IQR 3–17]). The mean serum phosphate, calcium, and PTH levels were 1.90 (0.6) mmol/L, 2.29 (0.2) mmol/L, and 631 (700) ng/L.

**Table 1:** Cohort characteristics (*n* = 54)

Age (years)	49.0 (12)
African American (n, %)	33 (54)
Hispanic (n, %)	16 (30)
Women (n, %)	21 (39)
Diabetic (n, %)	34 (63)
Taking cinacalcet (n, %)	18 (33)
Taking calcium containing phosphate binder (n, %)	30 (56)
Taking non-calcium containing phosphate binder (n, %)	22 (41)
Receiving intradialytic vitamin D agonist	43 (80)
Taking angiotensin converting enzyme inhibitor (n, %)	20 (37)
Taking angiotensin receptor blocker (n, %)	11 (20)
Taking beta adrenergic receptor antagonist (n, %)	41 (76)
Serum calcium (mmol/L)	2.29 (0.2)
Serum parathyroid hormone (ng/L)	631 (700)
Serum phosphate (mmol/L)	1.9 (0.6)
Serum albumin (g/L)	38.2 (3)
Protein catabolic rate	1.03 (0.3)
Blood urea nitrogen (mmol/L)	20.2 (7.2)
Serum creatinine (µmol/L)	911 (250)
Serum potassium (mmol/L)	4.88 (0.6)
Kt/V	1.46 (0.2)
Hemoglobin (g/L)	105 (11)
Treatment Time (minutes)	233 (19)
Blood flow (mL/min()	409 (86)
Dialysate flow (mL/min)	683 (110)
Receiving 2 K bath (n, %)	45 (83)
Receiving 2.5 Ca bath (n, %)	53 (98)
Estimated dry weight (kg)	85.8 (21)
Percentage of interdialytic weight gain	3.00 (1.9)
Ultrafiltration rate (mL/kg/hr)	8.00 (3.7)
Pre-HD supine systolic BP (mmHg)	164 (27)
Pre-HD seated systolic BP (mmHg)	158 (21)
Post-HD seated systolic BP (mmhg)	142 (21)
Post-HD supine systolic BP (mmHg)	152 (21)
Pre-HD supine diastolic BP (mmHg)	91.5 (17)
Pre-HD seated diastolic BP (mmHg);	86.1 (16)
Post-HD seated diastolic BP (mmHg);	78.4 (15)
Post-HD supine diastolic BP (mmHg)	86.4 (14)
Mean ambulatory systolic BP (mmHg);	144 (14)
Mean ambulatory diastolic BP (mmHg);	79.9 (12)
Pre-HD TPRI (dynes*sec/cm^5^/m^2^)	3220 (850)
Plasma ET-1 (pg/mL)	2.42 (1.6)
Plasma ADMA (µmol/L)	0.76 (0.2)

*HD* Hemodialysis, *BP* Blood pressure, *TPRI* Total peripheral resistance index, *ET-1* Endothelin-1, *ADMA* Asymmetric dimethylarginine

### Blood pressure outcomes

**Fig. 1:**
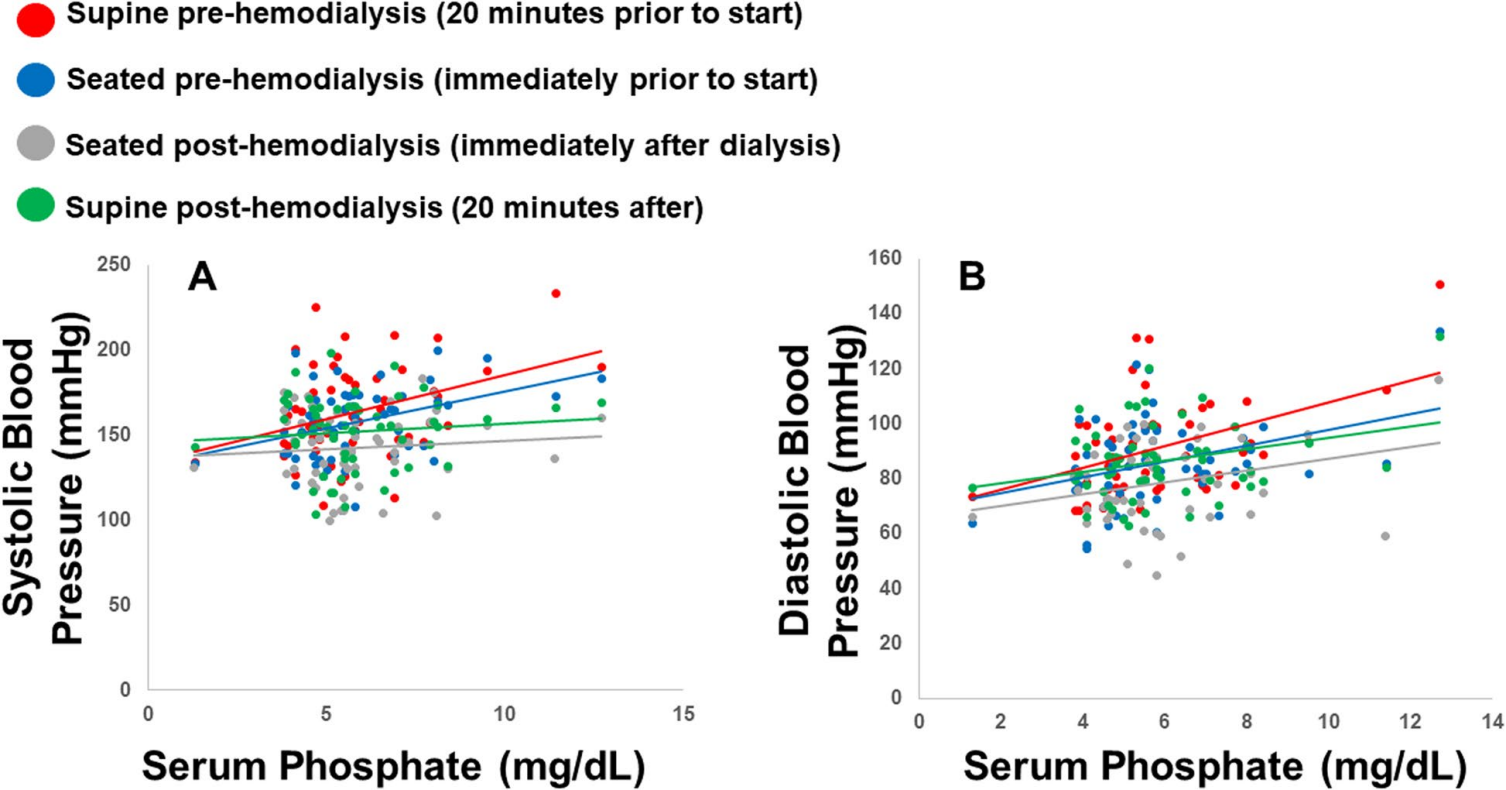
Scatterplot and trendlines for comparison of systolic (1A) and diastolic (1B) blood pressure vs serum phosphate. **A** shows the scatterplots and trend lines for systolic BP (y-axis) and serum phosphate (x-axis). The systolic BP measurements include supine measurements 20 min before dialysis in red (*r* = 0.3, *p* = .04), seated measurements immediately before HD in blue (*r* = 0.4, p = .002), seated measurements immediately after HD in gray (*r* = 0.08, *p* = .6), and supine measurements 20 min after dialysis in green (*r* = 0.09, *p* = .5). **B** shows the scatterplots and trend lines for diastolic BP (y-axis) and serum phosphate (x-axis). The diastolic BP measurements include supine measurements 20 min before dialysis in red (*r* = 0.5, *p* < .0001), seated measurements immediately before HD in blue (*r* = 0.5, *p* = .0003), seated measurements immediately after HD in gray (*r* = 0.4, *p* = 0.003), and supine measurements 20 min after dialysis in green (*r* = 0.4, *p* = .002)

The pre-HD and post-HD BP in the supine and seated positions are in Table 1. The scatterplots and trend lines showing the relationship between serum phosphate and the systolic and diastolic BP are shown in Fig. 1. There were significant correlations between serum phosphate with systolic BP for the pre-HD supine (Pearson *r* = 0.3, *p* = 0.04; Spearman *ρ* = 0.4, *p* = 0.004) and seated (*r* = 0.4, *p* = 0.002; Spearman *ρ* = 0.4, *p* = 0.004) measurements, but not for the post-HD seated (*r* = 0.08, *p* = 0.6; Spearman *ρ* = -0.01, *p* = 0.9) or supine (*r* = 0.09, *p* = 0.5; Spearman *ρ* = 0.3, *p* = 0.8) measurements. There were significant correlations between serum phosphate and diastolic BP for the pre-HD supine (*r* = 0.5, p < 0.0001; Spearman *ρ* = 0.3, *p* = 0.03) and seated (*r* = 0.5, *p* = 0.0003; Spearman *ρ* = 0.3, *p* = 0.04) measurements as well as the post-HD seated (*r* = 0.4, *p* = 0.003; Spearman *ρ* = 0.3, *p* = 0.04) and supine (*r* = 0.4, *p* = 0.002; Spearman *ρ* = 0.2, *p* = 0.12) measurements.

Table 2 shows the results of the repeated-measures linear regression analysis of BP outcome variables. In our model controlling for demographic variables (model 1), we found positive associations between serum phosphate and diastolic BP (β = 4.22, *p* = 0.0002) and systolic BP (β = 3.82, *p* = 0.058), albeit attenuated significance in systolic BP. In the models that included PTH and percentage of interdialytic weight gain (model 2), the significant positive association persisted between phosphate and diastolic BP (β = 4.31, *p* = 0.0002) but remained a trend for systolic BP (β = 3.82, *p* = 0.06). The mean number of hours available where ambulatory BP was measured in the *subsequent* interdialytic period was 34.0 (10) hours. Serum phosphate had a significant correlation with the mean diastolic using Pearson correlation (*r* = 0.3, *p* = 0.02) but not in Spearman correlation (*ρ* = 0.2, p = 0.3) or in systolic (Pearson *r* = 0.05, *p* = 0.7; Spearman *ρ* = 0.3, *p* = 0.8) ambulatory BP.

**Table 2:** Repeated measures linear regression analysis using pre-hemodialysis supine blood pressure as the primary outcome variable (*n* = 54)

	Model 1		Model 2	
	Estimate (SE)	*p*-value	Estimate (SE)	*p*-value
**Systolic Blood Pressure**				
Intercept	150.0 (16)	< .0001	147 (17)	< .0001
Phosphate (mg/dL)	3.82 (2.0)	.06	3.82 (2.0)	.06
Parathyroid hormone (ng/L)	-	-	0.002 (0.003)	.9
Interdialytic Weight Gain as percentage of body weight	-	-	0.78 (1.0)	.5
**Diastolic Blood Pressure**				
Intercept	84.3 (9.7)	< .0001	85.4 (10)	< .0001
Phosphate (mg/dL)	4.22 (1.1)	.0002	4.31 (6.7)	.0002
Parathyroid Hormone (ng/L)	-	-	-0.002 (0.002)	.4
Interdialytic Weight Gain as percentage of body weight	-	-	0.16 (0.7)	.8

While not present, all models also controlled for age, sex, race, ethnicity, diabetes, and the subsequent BP measurements (pre and post-HD seated, and post-HD supine)

### Vasoconstriction and markers of endothelial cell dysfunction

The Pearson correlation coefficients for serum phosphate with pre-HD TPRI, ET-1, and ADMA were 0.3 (*p* = 0.01; Spearman *ρ* = 0.2, *p* = 0.08), 0.4 (*p* = 0.007; Spearman *ρ* = 0.2, *p* = 0.11), and 0.3 (*p* = 0.04; Spearman *ρ* = 0.2, *p* = 0.15), respectively. Table 3 shows that there were independent associations between serum phosphate and both higher TPRI and ET-1 (negative regression coefficient in the context of reciprocal transformation) in models that included demographic variables, PTH and weight gain, while there was no serum phosphate association with ADMA. Parathyroid hormone (PTH) was positively correlated with ADMA (*r* = 0.3, *p* = 0.01) in univariate Pearson correlation analysis, but its statistical significance was slightly attenuated in the Spearman correlation (*ρ* = 0.2, *p* = 0.09) and with the regression analysis (*p* = 0.06). In addition, we explored other models that including albumin, PCR, or calcium (data not shown) as additional covariates and found the associations between phosphate and all outcomes remained unchanged. Thus, the final model did not include these covariates guided by AIC and statistical significance.

**Table 3:** Linear regression models showing associations between serum phosphate with various pre-HD outcomes

	Total peripheral resistance index (log transformation)		Endothelin-1 (reciprocal transformation)		Asymmetric dimethylarginine (log transformation)	
	Estimate ($\pm SE)$	*p*-value	Estimate ($\pm SE)$	*p*-value	Estimate ($\pm SE)$	*p*-value
Serum Phosphate (mg/dL)	0.05 (0.02)	.005	-0.03 (0.02)	.047	0.02 (0.02)	.3

Models also controlled for age, sex, diabetes, Black race or Hispanic ethnicity, Parathyroid Hormone (PTH), and percentage of interdialytic weight gain

## Discussion

The principal finding of this study was that serum phosphate was associated with high HD-unit BP, vasoconstriction, and markers of ECD. Phosphate was independently associated with higher pre-HD diastolic BP, TPRI, and ET-1 while controlling for numerous demographic variables, although there was not an independent association between phosphate with ADMA. Overall, these findings demonstrate a novel relationship between MBD and cardiovascular disease in ESRD patients that may be independent of vascular calcification.

Serum phosphate is associated with increased morbidity and mortality in CKD patients [1, 10], though it is unknown whether BP is directly involved in this relationship. Intervention studies in both animals and healthy humans have demonstrated increases in BP following extended periods of high dietary phosphate intake [11, 12]. One observational study in pre-ESRD CKD patients showed a moderate correlation between serum phosphate and systolic BP which was predominantly found in the patients with diabetes (*n* = 30), but there was not adjustment for any other variables [13]. Another study in ESRD patients found that diastolic, but not systolic, BP (both measured pre-HD) was higher in patients in the highest tertile of serum phosphate [14]. Our study is novel in that we controlled for numerous demographic variables that have been associated with high pre-HD BP, we analyzed phosphate as a continuous variable, and we included analyses of vasoconstriction measurements and markers of ECD [9, 15].

Some of the proposed mechanisms to explain a relationship between phosphate and BP include increased arterial stiffness, increased renin–angiotensin–aldosterone system (RAAS) activity, and increased sympathetic nervous system (SNS) activity [11, 12, 16, 17]. The negative consequences of increased arterial stiffness include increased central aortic BP (which can contribute to left ventricular hypertrophy) and widened pulse pressure (which can contribute to coronary hypoperfusion.) Unfortunately, the structural changes from vascular calcification may persist even after serum phosphate is acutely brought under control. Our findings of the particularly strong association between phosphate and *diastolic* BP seem to deviate from the phenotype of isolated systolic hypertension that is often seen with increased arterial stiffness, which suggests that another mechanism may be responsible. As this was a post-hoc analysis from a previously conducted study, we did not have any assessment of RAAS or SNS activity to further evaluate possible explanations for relationship between phosphate and vasoconstriction. However, a large percentage of patients were receiving RAAS inhibiting drugs and/or beta adrenergic receptor antagonists.

Another mechanism proposed to explain the relationship between phosphate and cardiovascular disease is ECD. In an in vitro study*,* rat aortic ring cells exposed to a high phosphate medium showed significant decrease in dilation compared to those exposed to lower phosphate-containing medium [2]. The same investigators found that healthy humans ingesting a high phosphate meal experienced acute reduction in flow mediated vasodilation post-prandially which was inversely correlated with the serum phosphate level [2]. This study also reported an increase in PTH following phosphate ingestion, which was not accounted for in the analysis. In a community based population study, where only 7% had estimated glomerular filtration rates < 60 mL/min/1.73 m^2^, the investigators found an association between serum phosphate and microvascular dysfunction assessed with skin capillaroscopy [18]. Surprisingly, a cross sectional study in patients undergoing hemodialysis fistula placement found that a U-shaped curve defined the relationship between serum phosphate and FMD independent of BP, race, or presence of diabetes [19]. Parathyroid hormone and other variables related to MBD and nutrition were not taken into consideration, and roughly one third of patients in that study had pre-dialysis CKD. Of note, one clinical trial found that suppression of PTH with intravenous vitamin D analogues improved FMD in CKD patients, but this effect was blunted among patients with persistently high phosphate levels [20]. Collectively, these demonstrate that phosphate and/or additional MBD factors adversely influence ECD.

We were able to evaluate some relationship between phosphate and ECD by including analysis of plasma ET-1 and ADMA. We found that phosphate was independently associated with ET-1, an endothelial cell derived vasoconstrictor. A phosphate-induced increase in ET-1 has been previously observed in both human endothelial cells and rat models of CKD [21]. Our novel finding in humans warrants longitudinal research to determine if improving phosphate control lowers ET-1 and possibly TPRI and BP. We also found that serum phosphate and PTH were associated with ADMA, an endogenous inhibitor of nitric oxide synthase that is associated with cardiovascular morbidity and mortality in HD patients [5]. This is consistent with the findings of Coen et al. revealing phosphate and PTH are individually associated with ADMA in ESRD patients [22].

Overall, our findings demonstrate a significant relationship between serum phosphate, vasoconstriction, and ECD in HD patients. It will ultimately need to be determined whether improvement in serum phosphate alone would be sufficient to improve ECD and lower BP in this population. One small randomized trial in CKD IV patients found that treatment with the phosphate binder sevelamer improved FMD, but use of a calcium-containing binder calcium acetate did not [23]. Improvement in FMD was strongly associated with levels of a calcification inhibitor, fetuin A, bringing forth an additional player in the interaction between MBD and vascular health. In that study, the phosphate remained high after treatment and many patients with comorbidities found in CKD and HD patients (diabetes, coronary artery disease, smoking, and use of renin–angiotensin–aldosterone system inhibitors or statins) were excluded. Another trial in a more heterogeneous CKD population found no significant changes or between-group differences in pulse wave velocity, coronary artery calcium score, or reactive hyperemia index (to assess ECD) in the participants randomized to lanthanum, calcium acetate, or low phosphate diet [24]. These studies highlight the need for further research that comprehensively takes into account the numerous mediators of MBD on vascular function and BP.

Limitations to our study include its relatively small size and the inability to draw conclusions about causality due its observational nature. Furthermore, we used ET-1 and ADMA as biomarkers for ECD. Because this was a retrospective analysis, we did not have alternative measurements such as FMD available. Additionally, the serum phosphate and other HD lab measurements were obtained in the context of routine clinical care and did not occur on the exact same date as our BP and TPRI measurements. However, we used the most recent measurements of phosphate preceding our measurements that usually occurred within a 1–2 week period. Our study had numerous strengths related to the variables that we ascertained and controlled for in the analysis to establish the presence of an independent association.

## Conclusion

In conclusion, we found that there is a positive association between serum phosphate and peri-dialytic BP. The association with diastolic BP was particularly strong and was independent of PTH, interdialytic weight gain and other variables. We found that phosphate was also related to pre-HD vasoconstriction and ECD markers, although the association with ADMA was not fully independent of other factors. These findings require further investigation to determine the hemodynamic benefits of aggressive phosphate lowering in ESRD and even possibly in pre-ESRD chronic kidney disease. They also introduce the possibility of more targeted approaches to BP management among patients with refractory hyperphosphatemia. Such research will require comprehensive assessment of the numerous mediators of MBD.

## Data Availability

The datasets analyzed during the current study are available from the corresponding author on reasonable request.

## Abbreviations

MBD: Mineral Bone Disease
ESRD: End stage renal disease
HD: Hemodialysis
PTH: Parathyroid hormone
ECD: Endothelial cell dysfunction
ADMA: Asymmetric dimethyarginine
BP: Blood Pressure
TPRI: Total peripheral resistance index
NICOM: Non-invasive cardiac output monitor
TPR: Total peripheral resistance
MAP: Mean arterial pressure
CO: Cardiac output
PCR: Protein catabolic rate
ET-1: Endothelin-1
GLM: Generalized linear regression models
AIC: Akaike Information Criteria
RAAS: Renin angiotensin aldosterone system
SNS: Sympathetic nervous system
